# Correlates of Sub-Optimal Feeding Practices among under-5 Children amid Escalating Crises in Lebanon: A National Representative Cross-Sectional Study

**DOI:** 10.3390/children9060817

**Published:** 2022-06-01

**Authors:** Maha Hoteit, Carla Ibrahim, Danielle Saadeh, Marwa Al-Jaafari, Marwa Atwi, Sabine Alasmar, Jessica Najm, Yonna Sacre, Lara Hanna-Wakim, Ayoub Al-Jawaldeh

**Affiliations:** 1 Faculty of Public Health, Section 1, Lebanese University, Beirut 6573, Lebanon; daniellesaadeh@hotmail.com (D.S.); marwaaljaafari@outlook.com (M.A.-J.); marwa.atwy@gmail.com (M.A.); sabine98asmar@gmail.com (S.A.); jess_najm98@hotmail.com (J.N.); 2 PHENOL Research Group (Public HEalth Nutrition prOgram Lebanon), Faculty of Public Health, Lebanese University, Beirut 6573, Lebanon; carla.t.ibrahim@outlook.com; 3 Lebanese University Nutrition Surveillance Center (LUNSC), Lebanese Food Drugs and Chemical Administrations, Lebanese University, Beirut 6573, Lebanon; 4 Department of Nutrition and Food Sciences, Faculty of Arts and Sciences, Holy Spirit University of Kaslik (USEK), P.O. Box 446, Jounieh 1200, Lebanon; yonnasacre@usek.edu.lb; 5 Faculty of Public Health, Section 2, Lebanese University, Beirut 6573, Lebanon; 6 INSPECT-LB (National Institute of Public Health, Clinical Epidemiology, and Toxicology), Beirut 00961, Lebanon; 7 Department of Agricultural and Food Engineering, School of Engineering, Holy Spirit University of Kaslik (USEK), P.O. Box 446, Jounieh 1200, Lebanon; larahanna@usek.edu.lb; 8 World Health Organization Regional Office for the Eastern Mediterranean, Cairo 11371, Egypt; aljawaldeha@who.int

**Keywords:** feeding practices, under-5 children, malnutrition, crises, Lebanon

## Abstract

Sub-optimal feeding practices among under-5 children are the major drivers of malnutrition. This study aims to assess the prevalence of malnutrition and the factors affecting exclusive breastfeeding, bottle feeding, and complementary feeding practices among under 5 children amid the COVID-19 pandemic as well as the economic and the political crises in Lebanon. A nationally representative stratified random sample of mother–child dyads (*n* = 511) was collected from households using a stratified cluster sampling design. The survey inquired about infant’s feeding and complementary feeding practices using a valid questionnaire. Anthropometric measurements of the mother and child were collected. Multivariate logistic regression was conducted to explore the determinants associated with under-5 children’s practices. The prevalence of underweight, stunting, wasting, overweight and obese children was 0.5%, 8.4%, 6.7%, 16.8% and 8.9%, respectively. In total, among under-5 children, the prevalence of ever breastfeeding, exclusive breastfeeding, and bottle feeding at birth was 95.1%, 59.1% and 25.8%, respectively. Half the children in this study started solid foods between 4 and 6 months. Regression analysis showed that supporting breastfeeding at hospital (aOR = 8.20, 95% CI (3.03–22.17)) and husband’s support (aOR = 3.07, 95% CI (1.9–4.92)) were associated with increased breastfeeding odds. However, mother’s occupation (aOR = 0.18, 95% CI (0.55–0.58)) was inversely associated with breastfeeding practices. Male children (aOR = 2.119, 95% CI (1.37–3.27), mothers diagnosed with COVID-19 (aOR = 0.58, 95% CI (0.35–0.95)), and bottle feeding at hospital (aOR = 0.5, 95% CI (0.32–0.77)) were more likely to induce early initiation of solid foods at 4 months of age. This study demonstrated non-negligible rates of malnutrition, low prevalence of exclusive breastfeeding, and high rates of early introduction of formula feeding and solid foods among Lebanese under-5-children amid escalating crises.

## 1. Introduction

### 1.1. Prevalence of Breastfeeding

Sub-optimal breastfeeding and poor complementary practices among infants and young children and sub-optimal access to nutritious food among under-5 children are the major drivers of malnutrition, impaired cognitive ability, and poor school performance [1]. Breastfeeding is cost-effective; increasing breastfeeding rates can help reduce the prevalence of numerous illnesses and health issues, resulting in lower healthcare costs [2,3]. The World Health Organization (WHO) and the United Nations International Children’s Emergency Fund (UNICEF) recommend starting breastfeeding within one hour of birth, breastfeeding exclusively for the first six months of life, and introducing nutritionally adequate complementary (solid) foods at six months, with continued breastfeeding for two years or longer [4]. The WHO originally established a global goal of 50% exclusive breastfeeding prevalence by 2025 [4]. It has been upgraded to at least 70% prevalence by 2030 [4]. According to a recent geographic analysis of exclusive breastfeeding prevalence estimates across 94 low- and middle-income countries (LMICS) from 2000 to 2018, the total prevalence of exclusive breastfeeding increased from 27% to 39% across all countries (2000–2018) [5]. By 2025, it is expected to increase to 43%. [5]. On the other hand, another published data from 57 low- and middle-income countries (LMICS) showed that the global weighted prevalence for early initiation of breastfeeding (EIBF) was 51.9%, that for exclusive breastfeeding under 6 months (EBF < 6 m) was 45.7%, that for continued breastfeeding at 1 year was 83.1% and that for continued breastfeeding at 2 years was 56.2% [6]. Among 57 LMICs, Eastern Mediterranean (34.5%), European regions (43.7%) and upper middle-income countries (38.4%) had poorer performance of EBF < 6 m [6]. Moreover, between 2000 and 2018, the Eastern Mediterranean region (EMR) faced a decline of 5.3% in exclusive breastfeeding rates under 6 months [6]. Although this could be positive progress at the global level and negative one at the EMR, both fall short of the 70% goal [6]. Nowadays, during the COVID-19 pandemic, international agencies encouraged women to continue breastfeeding. Despite attempts to limit the harmful promotion of breastmilk substitutes, nations are still failing to safeguard parents from false information, according to new research by the WHO, UNICEF, and the International Baby Food Action Network (IBFAN) [7].

### 1.2. Complementary Feeding Practices

As for the complementary feeding patterns, despite differences in current recommendations addressing when to start complementary solid foods, all of them agree to not introduce complementary feeding before the age of 6 months [7]. Appropriate complementary foods and feeding practices contribute to child survival, growth, and development; they can also prevent obesity, micronutrient deficiencies and undernutrition later in life [8]. According to the Global UNICEF database (2021), the global prevalence for the introduction of solid, semi-solid or soft foods under 6 months is 73%, and that at the EMR level is 68% [9]. Moreover, the complementary foods consumed by two out of three children are deficient in nutritious foods such as fruits, vegetables, legumes, nuts, and foods of animal origin such as eggs, fish, dairy, or meat [8]. In addition, young children are increasingly consuming nutrient-poor snack foods and beverages, which may predispose them to malnutrition [8].

### 1.3. Prevalence of Malnutrition

Malnutrition is responsible for over half of all deaths in under-5 children; it exposes children at risk of dying from common diseases, increases the frequency and severity of infections, and slows recovery [10]. The interplay of malnutrition and infection can result in a potentially fatal cycle of declining health and nutritional state. We are still a long way from a world free of hunger. Meanwhile, the UNICEF–WHO–World Bank Group Joint Malnutrition Estimates for 2021 reveal that stunting prevalence has been dropping since 2000; more than one in five under-5 children—149.2 million—were stunted in 2020, with 45.4 million suffering from wasting [11]. In the meantime, the number of under-5 children who are overweight has risen from 33.3 million in 2000 to 38.9 million in 2020 [11]. Before the COVID-19 pandemic, chronic childhood malnutrition remained a major challenge in the EMR, including Lebanon, which has been known to have low rate of breastfeeding [12] of which only about 14.8% of infants under the age of six months experienced exclusive breastfeeding in 2015 [13,14].

### 1.4. Justification and Purpose of the Study

Children in Lebanon are currently facing the impact of one of the world’s biggest economic failures in recent time. The protracted economic crisis is just one of Lebanon’s multiple crises, which include the COVID-19 pandemic, the consequences of the enormous August 2020 Beirut Port explosions, and the country’s ongoing political instability. While the 1.5 million Syrian refugees are the most vulnerable, the number of Lebanese who require assistance is rapidly increasing [15]. None of the previous studies conducted in Lebanon focused on the prevalence of malnutrition and correlates of sub-optimal breastfeeding, poor complementary practices, and unhealthy feeding patterns among under-5 children in Lebanon amid the escalating crises. To guarantee successful promotion tactics, it is critical to understand the context-specific patterns and correlates of breastfeeding and complementary feeding practices. Thus, it is an ideal time to consider the impact of consecutive crises in Lebanon on feeding patterns and malnutrition among under-5 children. The purpose of this study is to retrospectively (1) estimate the prevalence of ever breastfeeding, exclusive breastfeeding, bottle feeding, continued breastfeeding, mixed milk feeding, and complementary feeding, (2) examine the factors associated with breastfeeding and complementary feeding practices, and (3) investigate the prevalence of wasting, stunting, underweight, and overweight in the under-5 offspring living in a nationally representative sample of Lebanese mother–child dyads.

## 2. Materials and Methods

### 2.1. Study Design and Sampling Technique

This study was carried out between January and December 2021. It included both the cross-sectional and retrospective design. A representative stratified random sample of children aged 5 years and younger of both genders (*n* = 511) was collected from households using a stratified cluster sampling design. Participants were targeted from all eight provinces in Lebanon (Beirut, Mont Lebanon, North Lebanon, Akkar, Bekaa, Baalbeck/Hermel, South Lebanon, and Nabatieh). The clusters were chosen at the district level, and the strata were the eight Lebanese governorates. Households were chosen from each district using a probability proportional to size technique, with more participating households recruited from more populous districts. In Lebanon’s districts, housing units were the major sample unit. Due to the COVID-19 pandemic restriction in Lebanon, eligible mothers were interviewed through online self-administered questionnaire about sociodemographic characteristics of the household and maternal and child characteristics, including infant feeding patterns. Participation in this study was entirely voluntary, and participants were aware of the study’s objective prior to their participation. Mothers eligible to participate in this study were Lebanese, aged between 18 and 49 years, and they have children aged 5 years and younger. Yet, every child aged more than 5 years and every non-Lebanese mother were excluded from this study.

### 2.2. Sample Size Calculation

A minimum participant sample size of 369 women of childbearing age was targeted to ensure appropriate power for statistical analyses to be carried out according to the Epi info [16] sample size calculations with a population size of Lebanese women of reproductive age of 1,551,344. The calculation of the sample size was conducted according to the latest population estimates of 2019–2020 and based on the Ministry of Public Health data [17], an alpha error of 5%, a power of 80% and a 40% expected frequency of infant formula consumption among children younger than 24 months of age in LMICs [18]. This number was, then, increased by almost 50% to reach a representative sample of women participants that takes the cluster effect and refusals into account; the final sample size comprised 511 participants, who were stratified according to Lebanese provinces to represent adequately the frequency of mother–child dyads.

### 2.3. Ethical Consideration

The study was conducted according to the guidelines of the Declaration of Helsinki and approved by the Ethics Committee of the Al-Zahraa University Medical Centre, Beirut, Lebanon, reference Nb 9-2020 (issued date: 2 December 2020). Each participant was given a thorough explanation of the study’s purpose and was assured of the data’s privacy and confidentiality. The estimated time for completion of the survey was 15–20 min. The participants were given the opportunity to ask any question via WhatsApp, messenger, and Zoom. The questionnaire was filled out by mothers and returned to the data collectors who were a team of trained dietitians. Participation was entirely voluntary. Mothers provided written informed consents.

### 2.4. Data Collection

#### 2.4.1. Assessment of Feeding Patterns and Their Correlates

A pre-tested online questionnaire was administered anonymously to mothers who consented to participate in this study. It was built in google form and posted on different sites of social media such as WhatsApp, Facebook, and Instagram. A validated questionnaire was retrieved from the Center of Diseases Control (CDC) website and adapted to the studied population taking into consideration the culture and the foods commonly consumed by the Lebanese under-5 children [19,20]. A focus group (*n* = 20) meeting was enrolled before launching the study to test the feasibility of all the questions. Information was collected on the following characteristics: (1) the mother, family members, and children’s COVID-19 status, (2) the mother’s weight, height, age, marital status, cooking, pregnancy status, current diet, breastfeeding status as a child, nationality, current residence, place of birth, income, employment status and work field, (3) the father’s educational level and work status, (4) regarding the household, the crowding index, the family income, (5) the number of children in the family, the age, height, and weight of each child, (6) the youngest child’s age, gender, place of delivery, weight, and height at birth, (7) the feeding practices, including breastfeeding, formula feeding, and complementary feeding, and (8) the knowledge, attitude, and practice towards breastfeeding (Knowledge: interval of exclusive breastfeeding, liquids fed to the infant, duration of breastfeeding, reasons to stop breastfeeding; Attitude and Practice: timing of breastfeeding post-delivery, first liquid supplied to the infant, information given in the hospital about breastfeeding, attitudes of hospital staff, doctors, paediatricians… towards the different methodologies of infant feeding, breastfeeding cessation; Social Support: from baby’s father, mother, mother-in-law, doctor or other health professional…). The percentage of children who were ever breastfed was also reported in this study. It was calculated according to the following formula: percentage of children 0–4.9 years of age who were ever breastfed = (children 0–4.9 years of age who were ever breastfed/children 0–4.9 years of age) × 100.

#### 2.4.2. Assessment of Malnutrition

**Figure 1:**
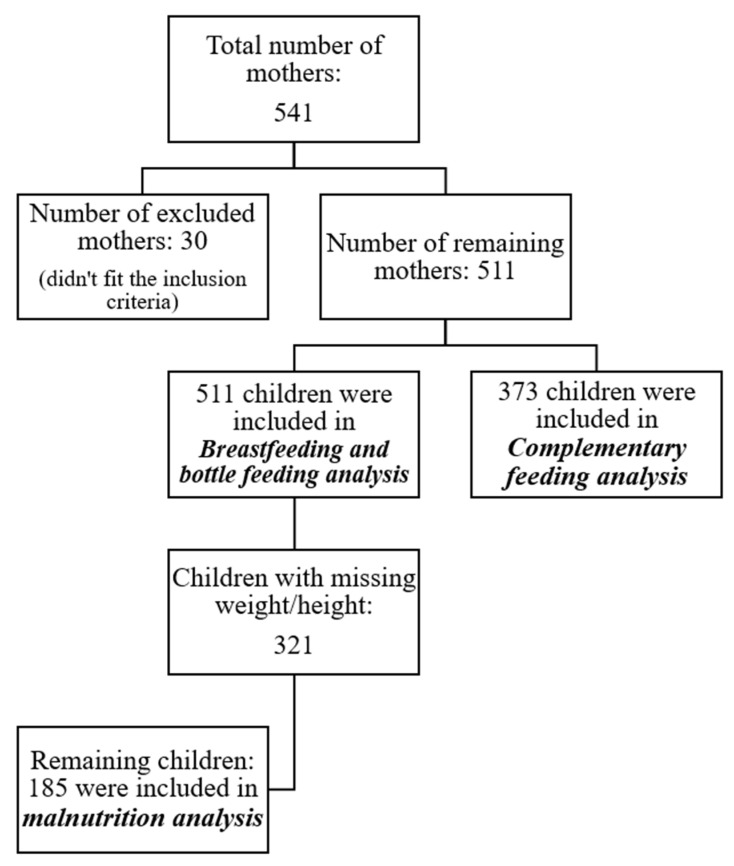
Description of the sampling details.

Anthropometric measurements of the mother and child were self-reported. Child’s height and weight data were derived from the latest record on the vaccination card (within 2 weeks to one month only). The restrictions imposed by the COVID-19 pandemic and the economic crisis worsen the access of mother–child dyads to healthcare premises and paediatrician’s clinics in Lebanon [21]. This was demonstrated through the children’s anthropometric measures reported in this study. Out of 511 children, a sub-sample of 185 children only were assessed for malnutrition (Figure 1). The WHO Anthro Survey Analyser [22] was used to convert weight, height, and age of child into height-for-age (HAZ), weight-for-age (WAZ), and weight-for-height (WHZ) Z-scores to assess stunting, underweight, wasting and overweight, respectively, taking the gender into consideration. Anthropometric classifications were based on 2006 WHO growth standards [23]: Stunted: HAZ < −2 SD to −3 SD; Wasted: WHZ < −2 SD to −3 SD; Overweight: WHZ > 2 SD; Underweight: WAZ < −2 SD to −3 SD. Figure 1 describes the sampling conducted in each analysis of this study.

### 2.5. Statistical Analysis

A “weighting” variable was created to adjust the over or under-representation of governorate in term of participants. Frequencies with percentages (%) and means with standard deviations (SD) were calculated to describe categorical and continuous variables, respectively. Chi-squared test, Pearson correlation and Student’s *t*-test were calculated, as appropriate, to compare infant feeding practices by sociodemographic, maternal, and child characteristics. Multivariate logistic regression models were computed to estimate the adjusted odds ratio (AOR) and 95% confidence intervals (CI) of EBF as well as the CF patterns and their odds. Moreover, further regression models were run, taking into account the variables related to COVID-19 (having been diagnosed with COVID-19 or having any of their family members or any of their children previously diagnosed with COVID-19) to investigate their associations with the dependent variables. Anthropometric indices were calculated using the WHO Anthro Survey Analyser. Prior to each analysis, participants with missing data were not selected (Figure 1). A *p*-value less than 0.05 was considered significant for all analyses. All statistical analysis was conducted using the Statistical Analysis Package for Social Sciences (SPSS, version 24.0), Armonk, NY, USA.

## 3. Results

### 3.1. Characteristics of the Study Population

Out of 541 recruited mother–child dyads, 511 were eligible to participate (response rate: 94.4%). The mean (±SD) age of recruited mothers was 30.2 ± 4.9 years. Half of the mother–child dyads lived in Beirut and Mount Lebanon. Only 5% of the study respondents stated having twins or triplets. Out of all respondents, 42.8% were currently working at the time of the data collection, and 25.5% of them were healthcare workers. The mean household crowding index was 1.03 ± 0.40. Of the total sample, 8.6% had low to no family income (less than 750,000 LBP) and 59.6% had a family income ranging between 750,000 and 2,250,000 LBP at the time of data collection. The mean (±SD) self-reported body mass index (BMI) of the mothers was 24.8 ± 4.5 kg/m^2^. More than half of the participants (52.7%) had a normal weight range (18.5 kg/m^2^ ≤ BMI < 25.0 kg/m^2^), 30% were overweight (25.0 kg/m^2^ ≤ BMI < 30.0 kg/m^2^) and 13.1% were obese (BMI ≥ 30 kg/m^2^). As for the COVID-19 infection, only 24.1% of the mothers have been diagnosed with COVID-19 (Table 1). Among the children, around half of them were female (54.8%) with a mean (±SD) age of 18 ± 15.5 months. Overall, the mean (±SD) weight and height at birth of the children was 3168.9 ± 0.617 kg and 49.5 ± 5.2 cm, respectively. Yet, male children were born with higher weight than their female counterparts (3283.35 ± 0.594 vs. 3074.18 ± 0.6222; *p* < 0.001). As for the COVID-19 infection, the highest proportion of children (83.6%) were having no history of COVID-19 infection; only 16.4% were diagnosed previously with COVID-19 (Table 2).

### 3.2. Prevalence of Malnutrition (Stunting, Wasting, Overweight and Underweight) and Feeding Patterns (Ever Breastfeeding, Exclusive Breastfeeding, Bottle Feeding, Continued Breastfeeding, Mixed Milk Feeding, and Introduction of Solid, Semi-Solid or Soft Foods)

More than half of the children aged under-5 years (*n* = 118) in this study were having a normal body weight (65.9%). However, 8.4% were stunted (HAZ < −2 SD to −3 SD) with a mean (±SD) height-for-age z-score of 0.4 ± 1.93. The prevalence of stunting in females was higher than in males (8.4% and 8.3%, respectively, *p*-value = 0.026). Additionally, 6.7% of the children were wasted (WHZ < −2 SD to −3 SD), 16.8% were overweight (WHZ > 2 SD), and 8.9% were obese (WHZ > 3 SD), with a mean (±SD) weight-for-height z-score of 0.5 ± 1.76. Furtherly, 0.5% of under-5 children were underweight (WAZ < −2 SD to −3 SD) with a mean (±SD) weight-for-age z-score of 0.6 ± 1.17 in both genders (*p*-value = 0.028). These findings are presented in (Table 3).

The prevalence of ever breastfeeding, exclusive breastfeeding, bottle feeding, continued breastfeeding, mixed milk feeding, and introduction of solid, semi-solid or soft foods among children aged between 0 and 59 months is shown in Table 3. Around 95.1% of under-5 children had ever been breastfed, 59.1% had EBF < 6 m, 19% had CBF for more than 1 year. On the other hand, among our study population, 55.8% had bottle feeding. Hence, the prevalence of bottle feeding at birth was 25.8%, at less than 1 month was 20.1%, at less than 6 months was 13.6%, 7.3% between 6 and 12 months, and at more than 12 months, it was 7.1%. Moreover, the prevalence of MixMF since birth was 29%. In addition, among the 372 respondents who started introducing solid, semi-solid or soft foods to their younger child, 47.1% of them introduced foods at 6 months, while 51.8% of them introduced foods between 4 and 6 months (of which 14.8% had introduced foods at 4 months). The introduction of solid, semi-solid or soft foods before 6 months of age was significantly more prevalent among males than females (46.1% vs. 30.1%, *p*-value = 0.008). On the other hand, the introduction of foods at 6 months of age was more prevalent in females (53.9% vs. 38%, *p*-value = 0.008) (Table 3).

### 3.3. Determinants of Breastfeeding, Bottle Feeding, and Complementary Feeding Practices

The determinants of breastfeeding, bottle feeding, and complementary feeding are presented in Table 4. Study findings show that mothers residing in Mount Lebanon and Beirut (*p* = 0.005), mothers working as healthcare workers (*p* = 0.003), mothers breastfed as a baby (*p* = 0.001), supporting breastfeeding at hospital (*p* < 0.001), paediatrician’s support (*p* = 0.034), husband’s support (*p* < 0.001), and family’s breastfeeding support (*p* < 0.001) were associated with positive breastfeeding practices (Table 4). As for the EBF duration, low family income (between 750,000 and 2,250,000 LBP) (*p* = 0.010) and mothers having twins or triplets (*p* = 0.002), were significantly associated with lower duration of exclusive breastfeeding less than 6 months. Furthermore, despite the husband’s and families’ support, it was observed that husbands with university educational levels (*p* = 0.001), husbands and or families who favoured breastfeeding over bottle feeding (*p* < 0.001, *p* = 0.009, respectively) were negatively affecting the breastfeeding duration (Table 4). On the other hand, the use of bottle feeding was associated with being child aged more than 6 months (*p* < 0.001), being a working mother (*p* = 0.015), being a healthcare worker mother (*p* = 0.002), mothers having twins or triplets (*p* = 0.001), mothers with COVID-19 history (*p* = 0.05) and having the family favouring breastfeeding (*p* = 0.025) (Table 4). However, there was no bottle feeding use when husbands were supporting breastfeeding (*p* < 0.001) (Table 4). Regarding complementary feeding, the early initiation of solid foods at 4 months was more prevalent in male children (*p* = 0.001), in children fed infant formula at hospital (*p* < 0.001), and among children with mothers infected with COVID-19 (*p* = 0.042) (Table 4).

### 3.4. Multivariate Analyses

The results of the multiple logistic regressions for the association between each feeding pattern (separate models for each) and sociodemographic and behavioural factors are presented in Table 5 In the multivariate logistic regression analysis, only variables that were determined to have statistically significant effects on breastfeeding, duration of exclusive breastfeeding, bottle feeding, and age of introduction of solid, semi-solid, or soft foods in the chi-squared test were included. In the first model (M1), when considering breastfeeding as dependent variable, results showed that mothers who were breastfed as a baby (aOR = 4.43, 95% CI (1.9–10.33)), family’s support (aOR = 5.46, 95% CI (2.34–12.75)), husband’s support (aOR = 8.84, 95% CI (3.38–23.10)), and breastfeeding practices at hospitals (aOR = 8.20, 95% CI (3.03–22.17)) were more likely to affect positively breastfeeding patterns. However, being a working mother (aOR = 0.18, 95% CI (0.55–0.58)) and living in a poor area (North and Akkar governorates) (aOR = 0.31, 95% CI (0.13–0.71)) were negatively affecting breastfeeding habits (Table 5, Model 1). In the second model (M2), when considering exclusive breastfeeding duration as a dependent variable, mothers who had their partners favouring breastfeeding instead of bottle feeding (aOR = 3.07, 95% CI (1.9–4.92)) were three times more likely to exclusively breastfeed their children for less than 6 months. In addition, mothers having twins or triplets (aOR = 0.14, 95% CI (0.29–0.71)) were less likely to exclusively breastfeed their children for 6 months and more (Table 5, Model 2). In the third model, when taking bottle feeding as the dependent variable, results showed that mothers with the husband both favouring breastfeeding instead of BOT (aOR = 0.39, 95% CI (0.18–0.84)), and those who had babies with older age (>6 months) who are fully weaned (aOR = 0.007, 95% CI (0.001–0.084)) were less likely to bottle feed (Table 5, Model 3). In the fourth model, taking the age of introduction of solid, semi-solid or soft foods as the dependent variable, it was observed that many factors were significantly associated with the early introduction of solid foods at 4 months. These correlates were: being a male child (aOR = 2.119, 95% CI (1.37–3.27)), being a child who was bottle-fed at hospital, and mother with COVID-19 history. In fact, the early introduction of solid, semi-solid or soft foods before 6 months of age was two times higher among boys than girls (aOR = 2.119, 95% CI (1.37–3.27)). Moreover, mothers who have been diagnosed with COVID-19 (aOR = 0.58, 95% CI (0.35–0.95)) and those whose children were bottle-fed at the hospital (aOR = 0.5, 95% CI (0.32–0.77)) were more likely to introduce complementary foods before 6 months (Table 5, Model 4).

## 4. Discussion

In the midst of escalating crises in Lebanon, the current study examines the prevalence of malnutrition and the factors affecting exclusive breastfeeding, bottle feeding, and complementary feeding patterns among under-5 children. Our data revealed significant malnutrition rates, low prevalence of exclusive breastfeeding, and high rates of early introduction of formula feeding and solid foods among under-5 children. Underweight, stunted, wasting, overweight, and obese children accounted for 0.5%, 8.4%, 6.7%, 16.8%, and 8.9% of the population, respectively. In sum, 95.1% of under-5 children had ever breastfed, 59.1% had exclusive breastfeeding <6 months, and 25.8% had bottle feeding at birth. Half of the children in this research study started solid foods before the age of 6 months. Additionally, many factors affected positively the breastfeeding practices, including breastfeeding support at hospital and husband’s support. However, being a working mother was inversely associated with improved breastfeeding practices. As for complementary feeding practices, male children, mothers diagnosed with COVID-19 and bottle feeding at hospital were the main factors that induce the early initiation of solid foods at 4 months of age.

### 4.1. Breastfeeding and Bottle-Feeding Practices

#### 4.1.1. Comparison with National Data

Compared to previous national studies conducted before the series of mutually reinforcing crises, our study revealed that despite the fact that Lebanese mothers were supported by their families and partners and were effective in initiating breastfeeding soon after birth, the rates of exclusive breastfeeding remained low. Moreover, the rates of early introduction of bottle feeding and early initiation of complementary feeding remained high. Nowadays, Lebanon’s severe recession has left families and children in a hazardous condition, affecting almost every facet of their life and well-being, with minimal resources and almost no access to healthcare facilities [24]. According to a recent survey conducted by UNICEF, more than 30% of children slept hungry and skipped meals in the month preceding the survey [24]. Moreover, 77% of households lack sufficient food or funds to purchase food, and 60% of households are forced to buy food on credit or borrow money. Furthermore, 40% of children are from families where no one works, and 77% are from families that do not receive any type of social support. A total of 80% of caregivers reported that their children struggled to concentrate on their studies at home—which could suggest hunger or mental distress [24].

Our findings in relation with breastfeeding practices were in line with the results reported in the national SMART survey (2021) in which 84.6% of children (6–23 months) were reportedly having ever been breastfed, 63% of newborns were initiated into early breastfeeding and the exclusive breastfeeding rates were 32.4% [25]. Similarly, our findings came hand in hand with the results of a recent cross-sectional Lebanese study that found that among under-5 children, the prevalence of exclusive breastfeeding at 40 days and at 6 months was 27% and 30%, respectively, and the prevalence of continued breastfeeding was 23%. The same study stated as well that around 60% of mothers breastfed their offspring between 0 and 6 month and half of them introduced infant formula at earlier stages between 0 and 6 months [26]. In comparison with other national studies, our findings show that there has been a higher prevalence of exclusive breastfeeding since 2015. According to a 2019 retrospective national cross-sectional survey, the average period of exclusive breastfeeding was 15 days, and the average age at which formula was administered was 2.03 months [27]. Exclusive breastfeeding began at 10.56 h on average, and half of the children had been exposed to formula milk since the first day after birth [27]. Moreover, another Lebanese cross-sectional study conducted between 2011 and 2012 stated that the rate of exclusive breastfeeding was 41.5% at 40 days and 12.3% at 6 months [28]. To add, the same study showed that at 40 days, 38.1% of the babies were mixed fed, whereas 20.2% were solely formula fed. At 6 months, 38.4% were mixed fed, while 40.1% received both bottle and solid feeding [28]. Furthermore, another study published in 2017 showed that around 40% of Lebanese newborns under the age of two months were exclusively breastfed, dropping to only 2% between the ages of four and five months. In fact, only 37.6% of newborns were given breastmilk as their first feeding after birth; other foods were given (infant formula, sweetened water, and herbal tea) [29]. Another cross-sectional study conducted in 2010 revealed that 55.9% of the mothers started breastfeeding within a few hours of birth, 18.3% within half an hour, and 21.2% within a few days; however, 4.6% of the moms did not breastfeed [30]. This study stated also that a considerable number of babies were fed liquids other than breast or formula milk earlier [30]. It is noteworthy that the prevalence of exclusive breastfeeding increased between 2015 and 2021 in Lebanon to reach 59.1%, thus exceeding the global and Middle eastern prevalence (44% and 44%, respectively) [31,32]. However, it remains far below the WHO recommendation of 70% [4]. On the other hand, among our study population, the prevalence of bottle feeding between 0 and 6 months was 59.5%; this rate was lower than the one reported in 2019 before the current escalating crises [26]. Moreover, the prevalence of mixed milk feeding since birth in our study was 29%, which is higher than the prevalence in the period preceding the devastating crises [26] but lower that that stated in the recent national SMART survey [25]. The increase in exclusive breastfeeding rates along with the decrease in bottle feeding could be due to the shortages of infant milk formula that are one facet of a food security challenge brought on by economic collapse and worsened by Lebanon’s reliance on imports for basic necessities such as fuel and wheat [33].

#### 4.1.2. Comparison with other EMR Countries

In comparison with other Arab countries, the prevalence of exclusive breastfeeding for 6 months in our study was higher than that of Saudi Arabia (27.6%) [34], Jordan (25.4%) [35], United Arab Emirates (16.9%) [36], Egypt (39.5%) [32], Iran (53.1%) [32], Iraq (25.8%) [32], Morocco (27.8%) [32], Oman (23.2%) [32], Palestine (38.9%) [32], Syrian Arab Republic (28.5%) [32], Tunisia (13.5%) [32], Yemen (9.7%) [32], and Algeria (28.6%) [32], yet, it was lower than that of Sudan (62.31%) [37] and similar to Qatar (60%) [38].

#### 4.1.3. Comparison with Other International Studies

Compared to some international regions and countries, our prevalence of exclusive breastfeeding for up to 6 months was higher than Spain (28.2%) [39], Nepal (23.2%) [40], Vietnam (18%) [41], and Ethiopia (48%) [41], and it was lower than the prevalence of exclusive breastfeeding in Bangladesh (72%) [41]. In total, according to UNICEF’s data, the prevalence of exclusive breastfeeding in the regions of the Americas (32%) [32], in the Western Pacific region (26%) [32], and in some countries of the Europe region [32]: Albania (36.5%), Belarus (21.7%), Montenegro (19.5%), Republic of Moldova (36.4%), Romania (15.8%), Serbia (23.6%), and Ukraine (19.7%), was below our findings. All these findings are shown in Table 6 All in all, national, regional, and international rates remain below the WHO new global target of exclusive breastfeeding (70%) by 2030 [4].

#### 4.1.4. Complementary Feeding Practices

Regarding complementary feeding practices in the current study, only 47.1% of mothers adhered to the WHO recommendations to introduce foods at 6 months. It is noteworthy that the prevalence of introducing solid foods at less than 6 months of age was lower than that reported in 2019 [26]. Currently, we found that 51.8% of the mothers introduced food between 4 and 6 months, from which 14.8% started introducing solid foods at the age of 4 months. However, in 2019, 60% of the mothers introduced complementary foods between 4 and 6 months, from which 40% started introducing solid foods before the age of 6 months [26]. In comparison with the findings reported by the national SMART survey (78.5%) [25], and that reported by UNICEF for the EMR (68%) [32], Jordan (83%) [35], and UNICEF at global level (73%) [32], the prevalence of introducing food at 6 months in our study was low (47.1%). On the other hand, our estimate prevalence of introducing food at <6 months (51.8%) was higher than that of the previous national study conducted in 2019 (40.6%) [26], Nepal (22.5%) [39], and Italy (14%) [42]. The pattern of early introduction of solid, semi-solid or soft foods may increase newborn morbidity and mortality due to decreased intake of protective substances found in breastmilk. In addition, mothers may produce less breastmilk after introducing solid foods; this may have an adverse effect on the infant’s nutritional intake. Furthermore, if complementary foods are not properly handled and stored, they may expose infants to hazardous pathogens [43].

#### 4.1.5. Correlates of Breastfeeding, Formula Feeding, and Complementary Feeding Practices

Breastfeeding and complementary feeding practices in Lebanon are hindered by the lack of highly qualified lactation specialists support, an inability to implement nationwide policies that encourage and protect breastfeeding practices, a poor social assistance, particularly at the family level, and many other sociodemographic factors [44].

In this study, the status of breastfeeding, bottle feeding, and complementary feeding among under-5 children in Lebanon was evaluated after adjusting for sociodemographic and behavioural determinants. Our findings stated that mothers who initiated breastmilk in the hospital were more likely to breastfeed their children. This aligns the results reported in a cross-sectional study published in 2018 in Abu Dhabi which found that babies who were receiving ready-to-use liquid formula in the hospital had a lower likelihood of exclusively breastfeeding [36]. Another Saudi study stated that the predominant inappropriate feeding practice seen in 76.2% of breastfeeding infants was the early introduction of formula at the hospital after birth [34]. These findings highlighted the conflicting effects of formula feeding on breastfeeding, which results in early termination of exclusive breastfeeding. Because of the inherent conflict of interest that hospitals and physicians frequently face between receiving extra funds from large food corporations and providing excellent care to their patients, the International Code of Marketing Breast-milk Substitutes and Lebanese law 47/2008 safeguard breastfeeding by banning marketing activities aimed at promoting breast milk substitutes. By enforcing international and national standards, the negative effects of baby formula advertising on breastfeeding success could be limited, preventing an increase in misinformation among women about the benefits of using infant formula. Despite the existence of several laws and international legislation protecting the value of breastfeeding, there is still a lack of implementation of these norms, particularly in Lebanon.

Furthermore, the current study stated that being a working mother was found to lower the duration of exclusive breastfeeding (less than 6 months of time). This result agreed with previous literature in which maternal employment was one of the barriers that causes early breastfeeding cessation [34,35,36,37,38,39,40,41,42,43,44,45]. Hence, working mothers were more likely to use infant formula, as revealed in our findings. It’s probable that full-time employed mothers with young children rely more heavily on bottle feeding options to help them deal with the busy pace of their life [46]. Additionally, returning to work, without proper support systems, has been shown to obstruct effective breastfeeding practices [46,47]. Breastfeeding support in the workplace is so critical for children’s health and development as well as for mothers’ and society’s overall well-being. Workplace breastfeeding rooms, paid nursing breaks, and a supportive breastfeeding environment are all low-cost treatments that can help increase breastfeeding, job productivity, and employee retention [47]. In regard to the husband’s role in promoting breastfeeding, Mannion et al. (2013) discovered that mothers who had a supportive partner felt more capable and competent while making breastfeeding decisions and facing problems [48]. This was supported in our study, where breastfeeding and exclusive breastfeeding for 6 months were positively associated with the family and husband preferring breastfeeding over infant formula [45]. Additionally, our findings showed that mothers having twins or triplets were less likely to exclusively breastfeed their children for 6 months and more. This is in line with a Turkish study where the prevalence of breastfeeding in twin babies was low [49]. Mothers of numerous babies believe they are poorly equipped to offer the best care for their children. Therefore, it is critical that mothers who are expecting twins or triplets be aware that their milk is sufficient; as well, they should be encouraged to breastfeed and should receive training on the benefits of breastfeeding [49]. Regarding determinants of complementary feeding, the introduction of solid, semi-solid or soft foods before the age of 6 months was much more common in boys than in girls. This finding was in line with previous research where boys were typically exposed to solid foods before girls [50,51,52]. A possible reason could be that male newborns consume more breast/formula milk, feed more often, and wake up more frequently at night. This may result in earlier weaning rather than supplementing with additional breast/formula milk [51,52,53,54]. Mothers who have been diagnosed with COVID-19 were more likely to introduce complementary foods to their children before 6 months of age. According to a cohort study conducted in Brazil, the introduction of complementary feeding before the 6th month of life occurs more likely among the interviewed women during the COVID-19 pandemic [55]. Furtherly, a case-control study conducted in Northeastern Italy found that women in the COVID-19 group had a 20% higher chance of introducing solid foods before 6-month [56]. An Indonesian study who examined complementary feeding behaviours during the COVID-19 outbreak discovered that maternal knowledge about complementary feeding in the pandemic environment is very crucial to its appropriate practice [57]. Mothers who have confirmed or suspected COVID-19 are concerned about passing the virus to their infants through breastmilk; thus, they tend to stop breastfeeding and introduce solid foods early [57]. However, even if they have confirmed or suspected being infected by COVID-19, the WHO and UNICEF advise mothers to continue breastfeeding during the COVID-19 pandemic [7]. While researchers continue to examine breastmilk from moms who have confirmed or suspected COVID-19, current evidence suggests that COVID-19 is unlikely to be transmitted by breastfeeding or giving breastmilk that has been expressed by a woman who has COVID-19 [7]. Hence, to build a mother’s competence to maintain appropriate breastfeeding and complementary feeding practices in any circumstance, awareness sessions and sustainable interventions in infant and young child feeding knowledge are required.

### 4.2. Malnutrition Data

The restrictions imposed by the COVID-19 pandemic and the economic crisis worsen the access of mother–child dyads to healthcare premises and paediatrician’s clinics in Lebanon [21]. This was demonstrated through the children’s anthropometric measures reported in this study. Our findings showed that more than half of the children aged under-5 years were having a normal body weight (65.9%). However, 8.4% were stunted (HAZ < −2 SD to −3 SD), 6.7% were wasted (WHZ < −2 SD to −3 SD), 16.8% were overweight (WHZ > 2 SD), 8.9% were obese (WHZ > 3 SD), and 0.5% were underweight (WAZ < −2 SD to −3 SD).

#### 4.2.1. Comparison with Other National Data

Concerning stunting’s prevalence, our findings (8.4%) were higher than the national SMART survey data (7%) [25], lower than the previously published national data of Abi Khalil et al. (9.3%) [26], and lower than UNICEF’s data (10.4%) [11]. On the other hand, concerning the prevalence of underweight children, our estimate prevalence (0.5%) was lower than that of the national SMART survey data (3.7%) [25] and previously published national data (9.3%) [26]. As for the wasting’s prevalence, our results (6.7%) were in line with old UNICEF’s data (2004) (6.6%) [11] and higher than the national SMART survey data (1.8%) [25] and the national data of 2019 (6.25%) [26]. Regarding overweight prevalence, our rate (16.8) was lower than UNICEF’s data (2004) (19.7%) [11] and the national data published recently (24.45%) [26], while it was higher than the national SMART survey data (3.7%) [25] and higher than the national data published in 2017 (6.5% in 2011–2012) [58]. Concerning the prevalence of obese under-5 children, our findings (8.9%) were higher than the national SMART survey data (1.2%) [25] and the national data published in 2017 (2.7% in 2011–2012) [58].

#### 4.2.2. Comparison with Other EMR Countries

Concerning stunting’s prevalence, our findings (8.4%) came hand in hand with Iran (8.45%) [59], while they were higher than Jordan (7.7%) [35], Kuwait (6%) [11], Bahrain (5.1%) [11], Qatar (4.6%) [11], and Saudi Arabia (3.9%) [11], and lower than the EMR’s and UNICEF’s data (26.2%) [11] and the regional average prevalence of stunting in 2018 (28%) [60]. In addition, in comparison with other regional countries, our findings were below Sudan (36.35%) [37], Pakistan (81.1%) [61], Yemen (47%) [62], Somalia (31%) [63], Egypt (22.3%) [11], Iraq (11.6%) [11], Oman (12.2%) [11], Syrian Arab Republic (29.6%) [11], Algeria (9.3%) [11], and Tunisia (8.6%) [11]. Concerning the prevalence of underweight children, our estimate prevalence (0.5%) was lower than Iran (7.63%) [59], Sudan (29.16%) [37], Jordan (3%) [35], Pakistan (57.3%) [61], and Yemen (39%) [62]. In total, our findings were below the EMR regional average prevalence of stunting in 2018 (18%) [60]. As for wasting’s prevalence, our results (6.7%) were higher than Jordan (2.4%) [35], while they were lower than the regional average prevalence of wasting in 2018 (8.69%) [60], the regional data reported by UNICEF (7.4%) [11], Iran (8.04%) [59], Sudan (13.6%) [37], Pakistan (18.2%) [61], Yemen (16%) [62], Somalia (21%) [63], and UNICEF’s data of Egypt (9.5%) [11], Oman (9.3%) [11], Saudi Arabia (11.8%) [11], Syrian Arab Republic (11.5%) [11] and Yemen (16.4%) [11]. Additionally, it was higher than UNICEF’s data of Algeria (2.7%) [11], Iraq (3%) [11], Kuwait (2.5%) [11], Qatar (2.1%) [11], Palestine (1.3%) [11], and Tunisia (2.1%) [11] and in line with UNICEF’s data of Bahrain (6.6%) [11]. Regarding overweight’s prevalence, our rate (16.8%) was higher than the regional UNICEF data (7.7%) [11], Iran (1.23% in 2018) [59], Jordan (4.7%) [35], Iraq (9%) [11], Kuwait (7.1%) [11], Oman (4.8%) [11], Saudi Arabia (7.6%) [11], Palestine (8.5%) [11], Yemen (2.7%) [11], Sudan (2.14%) [37], Algeria (12.9%) [11], Qatar (13.9%) [11], and Bahrain (6.4%) [11], and lower than Egypt (17.8%) [11], the Syrian Arab Republic (18.2%) [11], and Tunisia (16.5%) [11]. Concerning obesity’s prevalence, our findings (8.9%) were higher than Sudan (0.85%) [37], Jordan (4.7%) [35], and Iran (8% in 2017) [64]. In total, the regional average prevalence of overweight and obesity in 2018 (8.69%) [60] was lower than that reported in our study (12.85%).

#### 4.2.3. Comparison with Other International Studies

Concerning stunting’s prevalence, our findings (8.4%) were lower than the global data reported by UNICEF (22%) [11], the Regions of the Americas (8.9%) [11], the Western Pacific Region (9.3%) [11], Peru (14.4%) [65], and Brazil (12.7%) [66], and higher than the estimate prevalence reported in the European Region (5.7%) [11]. Reporting the underweight prevalence, our findings (0.5%) were lower than the global data reported by UNICEF (12.6%) [11], East Asia and Pacific region (5.18%) [11], and the Regions of the Americas (2.5%) [11]. As for wasting prevalence, our results (6.7%) were in line with the global UNICEF data (6.7%) [11], higher than the Region of the Americas (0.7%) [11], Peru (0.1%) [64], Brazil (5.1%) [65], and the Western Pacific Region (2.1%) [11]. As for overweight prevalence, our rate (16.8) was higher than the UNICEF data at the global level (5.7%) [11], as well, it was higher than the Region of the Americas (8%) [11], Brazil (12.6%) [64], the Europe Region (7.9%) [11], and the Western Pacific Region (7.5%) [11]. Finally, the prevalence of obesity in our findings (8.9%) was lower than that reported in Latin America and the Caribbean (9.2%) [67] and Central and Eastern Europe and Central Asia (10.9%) [67]. All these findings were described in Table 6.

**Table 6:** National, regional, and global prevalence of feeding patterns and malnutrition among under 5 years children.

Countries	Prevalence of Infant Feeding					Prevalence of Malnutrition					References
	EBF (%)	BOT (%)	CBF (%)	MixMF (%)	CF (%)	Stunting (%)	Under-Weight (%)	Wasting (%)	Overweight (%)	Obesity (%)	
Lebanon (Current study) (2021, during the crisis)	0–6 m: 59.1	At birth: 25.8 At <1 m: 20.1 At <6 m: 13.6 6–12 m: 7.3 At >12 m: 7.1	>12 m: 19	At birth: 29	At 4 m: 14.8 4–6 m: 37 At 6 m: 47.1	8.4	0.5	6.7	16.8	8.9	Current study.
Lebanon (2021)	0–6 m: 32.4	NA	12–23 m: 21.9	<6 m: 39	6–8 m: 78.5	7	3.7	1.8	3.7	1.2	[25]
Lebanon (2019, before the crisis)	At 40 d: 27 At 6 m: 30	Birth-40 d: 52.9 40 d-6 m: 21.9 At 4 m: 13.8 At >6 m: 10.7	>6 m: 23	At birth: 10.6	At <4 m: 1.3 4–6 m: 39.3 At 6 m:39.1	9.3	9.3	6.25	24.45	NA	[26]
Lebanon (2016)	At ≤1 m: 62.2 2–3 m: 20.83 4–6 m: 16.54	At 1 d: 51.6	>6 m: 0.4	NA	NA	NA	NA	NA	NA	NA	[27]
Lebanon (2011–2012)	At 40 d: 41.5 At 6 m: 12.3	At 40 d: 20.2	NA	At 40 d: 38.1 At 6 m: 38.4	At 6 m: 40.1	NA	NA	NA	NA	NA	[28]
Lebanon (2011–2012)	NA	NA	NA	NA	NA	NA	NA	NA	6.5	2.7	[58]
Lebanon (2010)	At <2: 40 4–5 m: 2	NA	NA	NA	NA	NA	NA	NA	NA	NA	[29]
Lebanon (2003–2004)	NA	NA	NA	NA	At 4 m: 41.6 At 5 m: 11.3 At 6 m: 13.4	NA	NA	NA	NA	NA	[30]
Lebanon (UNICEF database, 2004) *	NA	NA	12–23 m: 14.3	NA	NA	16.5	NA	6.6	16.7	NA	[32]
Lebanon (UNICEF/WHO/World Bank database, 2000–2020) *	NA	NA	NA	NA	NA	In 2000: 16 In 2020: 10.4	NA	NA	In 2000: 17.6 In 2020: 19.7	NA	[11]
EMR (UNICEF/WHO/World Bank database, 2000–2020) *	NA	NA	NA	NA	NA	In 2000: 33.8 In 2020: 26.2	NA	In 2020: 7.4	In 2000: 7.2 In 2020: 7.7	NA	[11]
Algeria *						In 2020: 9.3%		In 2019: 2.7	In 2020: 12.9%		
Egypt *						In 2020: 22.3		In 2014: 9.5	In 2020: 17.8		
Bahrain *						In 2020: 5.1		In 1995: 6.6	In 2020: 6.4		
Iraq *						In 2020: 11.6		In 2018: 3	In 2020: 9		
Kuwait *						In 2020: 6		In 2017: 2.5	In 2020: 7.1		
Oman *						In 2020: 12.2		In 2017: 9.3	In 2020: 4.8		
Qatar *						In 2020: 4.6		In 1995: 2.1	In 2020: 13.9		
Saudi Arabia *						In 2020: 3.9		In 2004: 11.8	In 2020: 7.6		
Syrian Arab Republic *						In 2020: 29.6		In 2010: 11.5	In 2020: 18.2		
Palestine *						NA		In 2020: 1.3	In 2020: 8.5		
Tunisia *						In 2020: 8.6		In 2018: 2.1	In 2020: 16.5		
EMR (UNICEF database, 2014–2020) *	0–5 m: 44	NA	12–23 m: 58	NA	6–8 m: 68	NA	NA	NA	NA	NA	[32]
Egypt (2014) *	39.5	-	-	-	-	-	-	-	-	-	
Iran (2010) *	53.1										
Iraq (2018) *	25.8										
Morocco (2017) *	27.8										
Oman (2017) *	23.2										
Palestine (2020) *	38.9										
Syrian Arab Republic (2019) *	28.5										
Tunisia (2018) *	13.5										
Yemen (2013) *	9.7										
Algeria (2019) *	28.6										
EMR (2018)	29.3	NA	NA	NA	NA	28	18	8.69	8.42		[60]
Iran (2018)	NA	NA	NA	NA	NA	8.45	7.63	8.04	1.23	NA	[59]
Saudi Arabia (2019)	0–6 m: 27.6	NA	For 2 y: 20.4	74.3	NA	NA	NA	NA	NA	NA	[34]
Sudan (2018–2019)	62.31	NA	Up to 2 y: 73.29	NA	NA	36.35	29.16	13.6	2.14	0.85	[37]
Jordan (2000–2018)	In 2017–2018: 0–5 m: 25.4	NA	In 2017–2018: 12–23 m: 26.1	NA	In 2017–2018: 6–8 m: 83	In 2012: 7.7	In 2012: 3	In 2012: 2.4	In 2012: 4.7		[35]
Rural setting, Pakistan (2017–2018)	NA	NA	NA	NA	NA	81.1	57.3	18.2	NA	NA	[61]
Qatar (2017)	1–3 m: 40 4–6 m: 20	NA	≥12 m: 34.8	NA	NA	NA	NA	NA	NA	NA	[38]
Iran (2017)	NA	NA	NA	NA	NA	NA	NA	NA	9	8	[64]
Abu Dhabi, United Arab Emirates (2014–2015)	0–6 m: 16.9	NA	NA	NA	NA	NA	NA	NA	NA	NA	[36]
Yemen (2013)	NA	NA	NA	NA	NA	47	39	16	NA	NA	[62]
Somalia (2007–2010)	NA	NA	NA	NA	NA	31	NA	21	NA	NA	[63]
Global (UNICEF database, 2000–2020) *	NA	NA	NA	NA	NA	In 2000: 33.1 In 2020: 22	NA	In 2020: 6.7	In 2000: 5.4 In 2020: 5.7	NA	[11]
Global (UNICEF/WHO/World Bank database, 2014–2020) *	0–5 m: 44	NA	12–23 m: 65	NA	6–8 m: 73	NA	NA	NA	NA	NA	[32]
Region of the Americas (2000–2020) *	In 2014–2020: 32	NA	In 2014–2020: 12–23 m: 31	NA	NA	In 2020: 8.9	NA	In 2020: 0.7	In 2020: 8	NA	[32]
Europe Region (2000–2020) *	NA	NA	NA	NA	NA	In 2020: 5.7	NA	NA	In 2020: 7.9	NA	[32]
Albania (2017) *	36.5										
Belarus (2019) *	21.7										
Montenegro (2018) *	19.5										
Republic of Moldova (2012) *	36.4										
Romania (2004) *	15.8										
Serbia (2019) *	23.6										
Ukraine (2012) *	19.7										
Western Pacific Region (2000–2020) *	In 2014–2020: 0–5 m: 26	NA	NA	NA	In 2014–2020: 6–8 m: 84	In 2020: 9.3	NA	In 2020: 2.1	In 2020: 7.5	NA	[32]
Latin America and the Caribbean (2015)	NA	NA	NA	NA	NA	NA	NA	NA	NA	9.2	[67].
Central and Eastern Europe and Central Asia (2015)	NA	NA	NA	NA	NA	NA	NA	NA	NA	10.9	[67]
Italy (2019)	At 3 m: 68	At 3 m: 17	NA	NA	At 3 m: 14	NA	NA	NA	NA	NA	[42]
Nepal (2017–2018)	0–6 m: 23.2	NA	NA	0–6 m: 48.6	<5 m: 22.5	NA	NA	NA	NA	NA	[40]
Peru (2009–2016)	NA	NA	NA	NA	NA	14.4	0.3	0.1	NA	NA	[65]
Spain (2014–2015)	At 6 m: 28.2	NA	NA	NA	NA	NA	NA	NA	NA	NA	[39]
Bangladesh Vietnam Ethiopia (2010–2014)	72 18 48	NA	NA	NA	NA	NA	NA	NA	NA	NA	[41]
Brazil (2009–2017)	NA	NA	NA	NA	NA	In 2009: 13.7 In 2017: 12.4	NA	In 2009: 5.7 In 2017: 5.1	In 2009: 11.6 In 2017: 12.6		[66]

EBF: Exclusive breastfeeding, BOT: Bottle feeding, CBF: Continued breastfeeding, MixMF: Mixed milk feeding, CF: Complementary feeding; NA: Not available, d: day, m: month, y: year. * In all UNICEF data, we referred to the point estimate value.

### 4.3. Summary

Our findings point to an advanced and critical stage of nutrition transition amid the crisis in Lebanon. Infant feeding practices in Lebanon fall short of international recommendations. Additionally, children are nowadays threatened in Lebanon. In general, children are particularly vulnerable in emergency situations. In as little as two weeks, child death rates can rise twenty-fold, reaching up to 70 times greater than in non-emergency situations [68]. During a crisis, the youngest children are the most vulnerable, especially if their feeding practices are poor to begin with. Infants and young children are at risk from living in overcrowded shelters, lack of access to nutritious food or clean water, lack of sanitation services, and difficulty navigating an overburdened health system. The protection, promotion, and support of excellent baby and young child feeding practices is a critical lifesaving intervention during emergencies and displacements [68]. Hence, this circumstance necessitates the development and/or revision of policies and programs, such as Infant and Young Child Feeding (IYCF) Counselling, as well as the Baby-Friendly Hospitals Initiative (BFHI), that have the potential to lower the risk and impact of malnutrition and sub-optimal infant feeding pattern. Additionally, a good support for the healthcare system should be prioritised. We hope that these findings will help public policymakers completely realise the country’s commitment to the UN Decade of Action on Nutrition and Sustainable Development Goal 2, which is to eliminate all types of malnutrition among under-5 children by 2025.

### 4.4. Limits and Strengths

The study had some limitations and strengths. Cause-and-effect correlations are not evident because the study is cross-sectional. In addition, there is a risk of recall bias, self-reporting inaccuracies, and social desirability bias due to the data collection techniques used. Moreover, using a small sub-sample size to assess the estimates of malnutrition may not represent the whole population. However, they can give us an approximation of the malnutrition status among under-5 years Lebanese children. This study, on the other hand, shows some strengths. It used a high sample size to ensure that the research questions were appropriately controlled and that the population profile was accurately represented. As well, the study offers a new and unique lens into infant feeding practices related to under-5 children amid the crisis in Lebanon.

## 5. Conclusions

This study gives an update on the feeding practices and the malnutrition status of Lebanese under-5 children, amid the COVID-19 pandemic, the economic and political crises. Although Lebanese mothers were successful in starting breastfeeding shortly after birth, rates of exclusive breastfeeding were low, and rates of early introduction of formula feeding were high. In addition, higher rates of early introduction of complementary feeding before six months were found among Lebanese infants. Furthermore, stunting, overweight, and obesity were more prevalent in children aged between 0 and 59 months. Thus, postnatal care in Lebanese health care facilities should be examined, and appropriate communication strategies for nutrition teaching should be established. In order to succeed in improving breastfeeding and complementary feeding rates and duration, policy implementation and enforcement of international legislations and national laws, as well as raising awareness and providing support and a friendly atmosphere for breastfeeding moms, should involve all stakeholders. As a result, increasing pre- and post-natal awareness programs and emphasising the beneficial and vital function of breastfeeding for both the mother and the child is very crucial. Future research should assess the prevalence of malnutrition in a bigger representative sample size of under-5 years children using anthropometric measurements and z-score calculations.

## Figures and Tables

**Table 1:** Characteristics of the mothers in this study.

**Characteristics of Mothers**	**Mean ± SD**
Age (years)	30.25 ± 4.98
Weight (kg)	66.40 ± 12.66
Height (m)	1.63 ± 0.06
BMI (kg/m^2^)	24.85 ± 4.53
Household crowding index	1.03 ± 0.40
**Characteristics of mothers**	***n*** **(%)**
Number of children (*n* = 511)	
1 child	241 (47.2)
2–3 children	238 (46.6)
>3 children	32 (6.2)
Marital Status (*n* = 511)	
Married	509 (99.6)
Divorced	2 (0.4)
Governorate (*n* = 511)	
Beirut and Mount Lebanon	288 (56.4)
North and Akkar	124 (24.2)
Beqaa and Baalbeck/Hermel	39 (7.6)
South and Nabatieh	60 (11.8)
Family income (*n* = 510)	
Less than 750,000 LBP	44 (8.6)
Between 750,000 LBP and 2,250,000 LBP	304 (59.6)
More than 2,250,000 LBP	162 (31.8)
Currently working (*n* = 511)	
Yes	219 (42.8)
No	292 (57.2)
Healthcare worker among working mothers (*n* = 219)	
Yes	56 (25.6)
No	163 (74.4)
Has twins or triplets (*n* = 511)	
Yes	26 (5)
No	485 (95)
Educational level of the spouse (*n* = 511)	
Illiterate	3 (0.6)
School level	233 (45.7)
University level	275 (53.7)
COVID-19 infection (*n* = 511)	
Yes	123 (24.1)
No	388 (75.9)

**Table 2:** Characteristics of under-5 years children in this study.

Under-5 Children	Overall *n* (%)	Female (54.8%) *n* (%)	Male (45.2%) *n* (%)	*p*-Value
Age (month) (Mean ± SD)	(*n* = 492)	(*n* = 271)	(*n* = 221)	0.312
	18.7 ± 15.5	18 ± 15.5	19.5 ± 15.6	
Weight at birth (g) (Mean ± SD)	(*n* = 507)	(*n* = 277)	(*n* = 230)	<0.001
	3168.9 ± 617.9	3074.18 ± 622.2	3283.35 ± 594.2	
Height at birth (cm) (Mean ± SD)	(*n* = 478)	(*n*= 260)	(*n* = 218)	0.312
	49.5 ± 5.2	49.2 ± 4.74	49.8 ± 5.7	
COVID-19 infection	(*n*= 510)	(*n* = 281)	(*n* = 229)	0.131
Yes	83 (16.4)	52 (18.6)	31 (13.7)	
No	427 (83.6)	229 (81.4)	198 (86.3)	

**Table 3:** Prevalence of malnutrition and feeding patterns among under-5 children in Lebanon.

Anthropometric Indices	Categories	Overall *n* (%) *	Female *n* (%)	Male *n* (%)	*p*-Value
HAZ	(Mean ± SD) *	0.4 ± 1.93	0.4 ± 2	0.4 ± 1.85	0.059
HAZ		(*n* = 179)	(*n* = 107)	(*n* = 72)	0.026
	Normal	158 (88.2)	92 (86)	66 (91.7)	
	Stunting (HAZ < −2 SD to −3 SD)	15 (8.4)	9 (8.4)	6 (8.3)	
	Severe Stunting (HAZ < −3 SD)	6 (3.4)	6 (5.6)	0 (0)	
WHZ	(Mean ± SD)	0.5 ± 1.76	0.4 ± 1.67	0.5 ± 1.89	1
WHZ		(*n* = 179)	(*n* = 105)	(*n* = 74)	0.093
	Normal	118 (65.9)	73 (69.5)	45 (60.7)	
	Wasting (HAZ < −2 SD to −3 SD)	12 (6.7)	7 (6.7)	5 (6.8)	
	Severe Wasting (HAZ < −3 SD)	3 (1.7)	2 (1.9)	1 (1.4)	
	Overweight (HAZ > 2 SD)	30 (16.8)	15 (14.3)	15 (20.3)	
	Obese (HAZ > 3 SD)	16 (8.9)	8 (7.6)	8 (10.8)	
WAZ	(Mean ± SD)	0.6 ± 1.17	0.6 ± 1.09	0.7 ± 1.27	0.028
WAZ		(*n* = 184)	(*n* = 109)	(*n* = 75)	0.795
	Normal	183 (99.5)	109 (100)	74 (98.7)	
	Underweight (HAZ < −2 SD to −3 SD)	1 (0.5)	0 (0)	1(1.3)	
Feeding patterns					
Ever breastfeeding		(*n* = 511)	(*n* = 281)	(*n* = 230)	0.226
Yes		486 (95.1)	270 (96.1)	216 (94.1)	
No		25 (4.9)	11 (3.9)	14 (5.9)	
Exclusive breastfeeding		(*n* = 486)	(*n* = 270)	(*n* = 216)	0.243
<6 months		287 (59.1)	166 (61.5)	121 (56.1)	
>6 months		199 (40.9)	104 (38.5)	95 (43.9)	
Continued breastfeeding		(*n* = 511)	(*n* = 281)	(*n* = 230)	0.034
>12 months		97 (19)	53 (18.9)	44 (19)	
Bottle feeding		(*n* = 511)	(*n* = 282)	(*n* = 229)	0.716
Yes		285 (55.8)	155 (55.1)	130 (56.7)	
No		226 (44.2)	127 (45)	99 (43.3)	
Initiation of bottle feeding		(*n* = 511)	(*n* = 281)	(*n* = 230)	0.073
At birth		132 (25.8)	68 (24.1)	64 (27.8)	
<1 month		102 (20.3)	64 (22.9)	38 (16.7)	
<6 months		70 (13.6)	34 (12)	36 (15.5)	
6–12 months		37 (7.3)	26 (9.3)	11 (4.7)	
>12 months		37 (7.1)	23 (8.1)	14 (5.9)	
No initiation of bottle feeding		133 (26.2)	66 (23.6)	67 (29.4)	
Feeding pattern since birth		(*n* = 511)	(*n* = 281)	(230)	0.415
Breastfeeding only		337 (65.9)	191 (67.9)	146 (63.5)	
Bottle feeding only		25 (5.1)	11 (4.1)	15 (6.3)	
Mixed milk feeding: breastfeeding + bottle feeding		148 (29)	79 (28)	69 (30.2)	
Introduction of solid, semi-solid or soft foods		(*n* = 373)	(*n* = 213)	(*n* = 160)	0.008
At 4 months		55 (14.8)	31 (14.4)	24 (15.4)	
4–6 months (6 not included)		138 (37)	64 (30.1)	74 (46.1)	
At 6 months		176 (47.1)	115 (53.9)	61 (38)	
>1 year		4 (1.1)	3 (1.6)	1 (0.5)	

* Mean ± SD is the mean value of each anthropometric index along with its standard deviation; *n* (%) is the population size along with its percentage.

**Table 4:** Factors associated with breastfeeding, bottle feeding, and complementary feeding.

Study Variables	Breastfeeding (*n* = 511)				Exclusive Breastfeeding (*n* = 486)				Bottle Feeding (*n* = 511)				Complementary Feeding (*n* = 373)			
	*n*	Yes (*n* = 486)	No (*n* = 25)	*p*-Value	*n*	<6 Months (*n* = 287)	≥6 Months (*n* = 199)	*p*-Value	*n*	Yes (*n* = 285)	No (*n* = 226)	*p*-Value	*n*	<6 Months (*n* = 193)	≥6 Months (*n* = 180)	*p*-Value
		*n* (%) +	*n* (%) +			*n* (%) +	*n* (%) +			*n* (%) +	*n* (%) +			*n* (%) +	*n* (%) +	
Gender of the younger children	511			0.305	486			0.266	285				372			**0.001**
Boy		216 (93.9)	14 (6.1)			121 (56.2)	94 (43.7)			130 (56.5)	100 (43.5)			98 (61.6)	61 (38.4)	
Girl		270 (96.1)	11 (3.9)			166 (61.3)	105 (38.7)			155 (55.2)	126 (44.8)	0.758		95 (44.6)	118 (55.4)	
Number of children	511			0.195	486			0.490								
1 or 2 child		390 (94.4)	23 (5.6)			233 (59.7)	157 (40.3)									
3 or more child		96 (98.0)	2 (2.0)			54 (56.2)	42 (43.8)									
Governorate	511			**0.005**	486			0.181	294			0.071	372			0.864
Beirut and Mount Lebanon		278 (96.5)	10 (3.5)			162 (58.3)	116 (41.7)			157 (54.5)	131 (45.5)			108 (51.2)	103 (48.8)	
North and Akkar		110 (89.4)	14 (10.6)			65 (59.1)	45 (40.9)			79 (56.5)	54 (43.5)			47 (51.6)	44 (48.4)	
Beqaa and Baalbeck/Hermel		39 (100.0)	0 (0.0)			29 (74.4)	10 (25.6)			29 (74.4)	10 (25.6)			16 (59.3)	11 (40.7)	
South and Nabatieh		36 (95.3)	24 (4.7)			31 (52.5)	28 (47.5)			29 (48.3)	31 (51.7)			21 (48.8)	22 (51.2)	
Family income	510			0.230	486			0.010	285			0.386	372			0.102
Less than 750,000 LBP		42 (95.5)	2 (4.5)			30 (71.4)	12 (28.6)			24 (54.5)	20 (45.5)			10 (34.5)	19 (65.5)	
Between 750,000 LBP and 2,250,000 LBP		286 (94.1)	18 (5.9)			178 (62.2)	108 (37.8)			177 (58.2)	127 (41.8)			112 (51.6)	105 (48.4)	
More than 2,250,000 LBP		158 (97.5)	4 (2.5)			79 (50.0)	79 (50.0)			84 (51.5)	79 (48.5)			71 (56.3)	55 (43.7)	
Education of the husband	511			0.725 ++	486			0.001 ++	285			0.059 ++	372			0.185 ++
Illiterate		3 (100.0)	0 (0.0)			1 (33.3)	2 (66.7)			3 (100.0)	0 (0.0)			2 (66.7)	1 (33.3)	
School level		221 (94.8)	12 (5.2)			149 (67.4)	72 (32.6)			139 (59.7)	94 (40.3)			92 (56.4)	71 (43.6)	
University level		263 (95.6)	12 (4.4)			137 (52.3)	125 (47.7)			143 (52.0)	132 (48.0)			98 (47.6)	108 (52.4)	
Mother currently working	219	206 (94)	13 (6)	0.344	206	127 (61.7)	79 (38.3)	0.318	219	136 (62.1)	83 (37.9)	**0.013**	92	56 (60.9)	36 (39.1)	0.159
Mother as healthcare worker	56	48 (85.7)	8 (14.3)	**0.003**	48	31 (64.6)	17 (35.4)	0.569	56	43 (76.8)	13 (23.2)	**0.002**	44	26 (59.1)	18 (40.9)	0.334
Mother has twins or triplets	26	23 (88.5)	3 (11.5)	0.128	23	21 (91.3)	2 (8.7)	0.002	25	22 (88.0)	3 (12.0)	**0.001**	19	9 (47.4)	10 (52.6)	0.815
Mother breastfed as a baby	406	393 (96.8)	13 (3.2)	**0.001 ++**	393	237 (60.3)	156 (39.7)	0.378	407	228 (56.0)	179 (44.0)	0.890	292	152 (52.1)	140 (47.9)	0.578
Paediatrician favoured breastfeeding	390	372 (95.4)	18 (4.6)	**0.034 ++**	373	216 (57.9)	157 (42.1)	0.497	390	208 (53.3)	182 (46.7)	0.111	287	146 (50.9)	141 (49.1)	0.917
Husband favoured breastfeeding	360	354 (98.3)	6 (1.7)	**<0.001**	354	186 (52.5)	168 (47.5)	<0.001	359	172 (47.9)	187 (52.1)	**<0.001**	256	132 (51.6)	124 (48.4)	0.918
Family supported breastfeeding	379	370 (97.6)	9 (2.4)	**<0.001**	370	206 (55.7)	164 (44.3)	0.009	379	200 (52.8)	179 (47.2)	**0.025**	269	132 (49.1)	137 (50.9)	0.132
Baby breastfed at hospital	483	465 (96.3)	18 (3.7)	**<0.001**	457	265 (58.0)	192 (42.0)	0.125	463	253 (54.6)	210 (45.4)	0.128	334	176 (52.7)	158 (47.3)	0.234
Mother with COVID-19 history	511	117 (95.1)	6 (4.9)	0.993	486	66 (56.4)	51 (43.6)	0.505	123	78 (63.4)	45 (36.6)	**0.05**	92	56 (60.9)	36 (39.1)	**0.041**
Age of the younger child									275			0.375	362			0.188
[0–6] months										72 (58.1)	52 (41.9)			40 (64.5)	22 (35.5)	
[6–12] months										56 (53.3)	49 (46.7)			47 (48.5)	50 (51.5)	
[1–3] years										94 (52.5)	85 (47.5)			72 (49.7)	73 (50.3)	
[3–5] years										53 (63.1)	31 (36.9)			29 (50.0)	29 (50.0)	
Baby age when stopping breastfeeding									274			**<0.001**	362			**0.050**
Less than 6 months										144 (91.1)	14 (8.9)			78 (58.6)	55 (41.4)	
6 months and more										130 (38.3)	209 (61.7)			109 (47.6)	120 (52.4)	
Baby fed infant formula at hospital													185	114 (61.6)	71 (38.4)	**<0.001**

+ Percentages are those of the total stated number for each factor; ++ Fisher’s Exact Test was used because more than 25% of the cells have expected count less than 5.

**Table 5:** The results of the multivariate analyses for the association of feeding pattern (breastfeeding, bottle feeding, and complementary feeding), and sociodemographic and behavioural factors.

Model 1. Logistic regression taking breastfeeding (Yes vs. No (reference)) as the dependent variable (*n* = 511)		
	aOR (95% CI)	*p*-value
Current residency (North and Akkar)	0.31 (0.13–0.71)	0.006
Mother breastfed as a baby	4.43 (1.90–10.33)	0.001
Healthcare worker mother	0.18 (0.55–0.58)	0.004
Family supporting breastfeeding	5.46 (2.34–12.75)	<0.001
Husband supporting breastfeeding	8.84 (3.38–23.10)	<0.001
Baby breastfed at hospital	8.20 (3.03–22.17)	<0.001
Variables entered in the model: Current residency, healthcare worker mother, mother breastfed as a baby, paediatrician supporting breastfeeding, husband supporting breastfeeding, family supported breastfeeding, baby breastfed at hospital		
Model 2. Logistic regression taking exclusive breastfeeding (<6 months (reference) vs. ≥ 6 months) as the dependent variable (*n* = 486)		
	aOR (95% CI)	*p*-value
Mother having twins or triplets	0.14 (0.29–0.71)	0.018
Husband supporting breastfeeding	3.07 (1.90–4.92)	<0.001
Variables entered in the model: Husband supporting breastfeeding, family supporting breastfeeding, family income, having twins or triplets, educational level of the husband.		
Model 3. Logistic regression taking the bottle feeding (Yes vs. No (reference)) as the dependent variable (*n* = 511)		
	aOR (95% CI)	*p*-value
Husband supporting breastfeeding	0.39 (0.18–0.84)	0.017
Baby age when fully weaned (>6 months)	0.007 (0.001–0.084)	<0.001
Variables entered in the model: Occupation of the mother, health care worker mother, husband supporting breastfeeding, baby age when fully weaned, family supporting breastfeeding, mother breastfed as a baby, mother with COVID-19 history.		
Model 4. Logistic regression taking the age of introduction of complementary foods (before 6 months (reference) vs. At 6 months and more) as the dependent variable (*n* = 373)		
	aOR (95% CI)	*p*-value
Baby gender (Male)	2.119 (1.37–3.27)	0.001
Baby fed infant formula at hospital	0.5 (0.32–0.77)	0.02
Mother with COVID-19 history	0.58 (0.35–0.95)	0.032
Variables entered in the model: Baby gender, baby fed infant formula at hospital, mother with COVID-19 history.		

aOR: Adjusted odds ratio; CI: confidence interval.

## Data Availability

Not applicable.
